# Effects of Medicare comprehensive medication review on racial/ethnic disparities in nonadherence to statin medications among patients with Alzheimer’s Disease: an observational analysis

**DOI:** 10.1186/s12913-022-07483-8

**Published:** 2022-02-07

**Authors:** Jamie A. Browning, Chi Chun Steve Tsang, Xiaobei Dong, Jim Y. Wan, Marie A. Chisholm-Burns, Christopher K. Finch, Jack W. Tsao, Colin Liu, Junling Wang

**Affiliations:** 1 Department of Clinical Pharmacy and Translational Science, University of Tennessee Health Science Center College of Pharmacy, 881 Madison Avenue, Memphis, TN 38163 USA; 2 Department of Preventive Medicine, University of Tennessee Health Science Center College of Medicine, 66 North Pauline St, Memphis, TN 38163 USA; 3 Children’s Foundation Research Institute, Le Bonheur Children’s Hospital, 50 North Dunlap St, Memphis, 38105 USA; 4 Department of Neurology, University of Tennessee Health Science Center College of Medicine, 855 Monroe Avenue, Memphis, TN 38163 USA; 5 University of Pennsylvania College of Arts and Sciences, Philadelphia, PA 19104 USA

**Keywords:** Comprehensive medication review, Medication therapy management, Alzheimer’s, Disparity, Race/ethnicity, Hyperlipidemia, Statins

## Abstract

**Background:**

Alzheimer’s Disease (AD) is the mostcommon cause of dementia, a neurological disorder characterized by memory loss and judgment impairment. Hyperlipidemia, a commonly co-occurring condition, should be treated to prevent associated complications. Medication adherence may be difficult for individuals with AD due to the complexity of AD management. Comprehensive Medication Reviews (CMRs), a required component of Medicare Part D Medication Therapy Management (MTM), have been shown to improve medication adherence. However, many MTM programs do not target AD. Additionally, racial/ethnic disparities in MTM eligibility have been revealed. Thus, this study examined the effects of CMR receipt on reducing racial/ethnic disparities in the likelihood of nonadherence to hyperlipidemia medications (statins) among the AD population.

**Methods:**

This retrospective study used 2015-2017 Medicare data linked to the Area Health Resources Files. The likelihood of nonadherence to statin medications across racial/ethnic groups was compared between propensity-score-matched CMR recipients and non-recipients in a ratio of 1 to 3. A difference-in-differences method was utilized to determine racial/ethnic disparity patterns using a logistic regression by including interaction terms between dummy variables for CMR receipt and each racial/ethnic minority group (non-Hispanic Whites, or Whites, as reference).

**Results:**

The study included 623,400 Medicare beneficiaries. Blacks and Hispanics had higher statin nonadherence than Whites: Compared to Whites, Blacks’ nonadherence rate was 4.53% higher among CMR recipients and 7.35% higher among non-recipients; Hispanics’ nonadherence rate was 2.69% higher among CMR recipients and 7.38% higher among non-recipients. Differences in racial/ethnic disparities between CMR recipients and non-recipients were significant for each minority group (*p* < 0.05) except Others. The difference between Whites and Hispanics in the odds of statin nonadherence was 11% lower among CMR recipients compared to non-recipients (OR = 0.89; 95% Confidence Interval = 0.85-0.94 for the interaction term between dummy variables for CMR and Hispanics). Interaction terms between dummy variables for CMR and other racial/ethnic minorities were not significant.

**Conclusions:**

Receiving a CMR was associated with a disparity reduction in nonadherence to statin medications between Hispanics and Whites among patients with AD. Strategies need to be explored to increase the number of MTM programs that target AD and promote CMR completion.

## Background

Alzheimer’s Disease (AD) is the most common cause of dementia, a neurological disorder characterized by memory loss and judgment impairment. AD affected an estimated 5.8 million Americans aged 65 years and older in 2020 [1]. In 2019 in the United States, AD was the sixth largest cause of death overall and the fifth largest cause of death among the 65 years and older population [2]. As life expectancy of the population extends in the US and the size of the elderly population grows, the prevalence of AD is expected to increase [3, 4]. Due to the high prevalence of AD in the US and the associated life-altering negative neurological symptoms, healthcare costs associated with AD are high and expected to increase. The estimated national healthcare cost of AD and other related dementias in 2020 alone was $305 billion and is predicted to increase to upwards of $1.1 trillion by 2050 [1, 5].

Representing 5-10% of dementia cases, dementia caused by cerebrovascular or vascular disease is the second most common type of dementia after AD, and such dementia frequently occurs in conjunction with AD [1]. This is considered a mixed pathology [1]. Research has been conducted to determine if hyperlipidemia could be a prominent risk factor for AD development. While the research community has yet to reach a consensus, recent studies have found a link between elevated lipids and AD [6–9]. Whether hyperlipidemia poses an increased risk for AD development or is simply a comorbid condition for some patients with AD, hyperlipidemia should be treated to reduce potential associated health complications among patients with AD and related dementias.

Adherence to medications to prevent potential adverse cardiovascular events is important in reducing health complications and healthcare costs associated with AD. However, medication adherence can be especially difficult for an individual with AD due to the complexity of AD management. Therefore, services offered by healthcare professionals to promote and increase medication adherence, such as medication therapy management (MTM), may be beneficial in this population.

In 2006, to combat medication utilization issues, the Centers for Medicare and Medicaid Services (CMS) began requiring MTM programs to be incorporated into Medicare prescription drug (Part D) plans [10]. The purpose of MTM programs is for pharmacists or other qualified healthcare providers to enhance therapeutic health outcomes for individuals by delivering patient-directed consulting services that aim to increase medication adherence, reduce adverse event risk, and improve medication utilization [11]. A major component of MTM programs is an annual Comprehensive Medication Review (CMR). A CMR is a consultation with the patient to interactively and thoroughly review their prescription and over-the-counter medications [12]. After a CMR, patients are provided with a printed summary of the consultation, including a Medication Action Plan with simple instructions and a Personal Medication List with all current medications and instructions [12]. A CMR grants MTM providers time to identify and solve medication-related issues while offering the recipient medication management advice and stressing the importance of medication adherence. Studies have shown that MTM services, such as CMRs, can reduce healthcare costs and improve medication adherence [13, 14].

Despite the evident benefits of MTM services and annual CMRs, there have been issues associated with the target populations for the MTM program. Each MTM program has liberty in determining MTM eligibility criteria under CMS guidelines, including which chronic conditions to target [15]. CMS requires that MTM programs target diseases on a list of pre-specified chronic conditions, but AD was not included as one of these until 2012 [15]. Nevertheless, AD has still not been a targeted condition by many MTM programs. For example, only 12.3% of MTM programs in 2017 included AD as a targeted condition [16]. Based on the most recent MTM program data available to researchers, only 14.3% of the MTM programs included AD in 2019 [17]. Furthermore, racial/ethnic disparities have been found to be associated with MTM eligibility [15, 18]. As a result, minority populations may be less likely than non-Hispanic Whites (Whites) to be eligible for MTM services. Consequently, CMS has broadened the MTM eligibility criteria for MTM programs in recent years with an intent to reduce disparities [15].

As MTM utilization data became available for research in recent years, an opportunity has arisen to examine the effects of MTM on disparities. Yet, no studies have examined the effect of CMRs on racial/ethnic disparities among the AD population. Therefore, this study aimed to determine if CMRs mitigate racial/ethnic disparities by reducing the likelihood of nonadherence to hyperlipidemia medications, specifically statins, among the AD population.

## Methods

This study retrospectively analyzed 2015-2017 Medicare data linked to Area Health Resources Files (AHRF) [19]. Medicare data included the Master Beneficiary Summary File (MBSF), Parts A/B claims, the Part D Event (PDE) File, and the Part D MTM Data File [20]. From these Medicare data files, beneficiary-level data were obtained. Specifically, the MBSF provided demographic and enrollment information for Medicare beneficiaries [21], and diagnosis information was obtained from Parts A/B data [20, 22]. Prescription utilization information, such as the drug name, service dates, and days supply, was provided by the PDE File [23], while MTM services information and CMR receipt were provided by the Part D MTM File [24]. Additionally, county-level information, such as healthcare capacity, income per capita, and education levels within the population, was obtained from AHRF [19]. The CMS Research Data Assistant Center facilitated access to the Medicare data utilized for this study.

The study sample included Medicare beneficiaries who met the following criteria in a study year: (1) aged 65 years or older; (2) were alive at the end of the study year; (3) had continuous Medicare Parts A, B, and D coverage; (4) had a diagnosis of AD based on the International Classification of Diseases version 9 (ICD-9) and version 10 (ICD-10) codes identified in medical claims from 2010 to 2017; and (5) met the inclusion/exclusion criteria that Pharmacy Quality Alliance (PQA) developed for calculating its adherence measure for statin medications. Based on the PQA criteria, individuals were included if they received at least two fills for statin medications on separate dates during the study period, with the first fill occurring 91 days prior to the end of the study period; patients were excluded if they had a diagnosis of end-stage renal disease or a record of hospice care [25, 26].

To examine the effects of CMRs, a Difference-in-Differences (DID) approach was used to compare the outcome differences across racial/ethnic groups between a treatment group (CMR recipients) and a control group (CMR non-recipients). The treatment group was composed of MTM enrollees who received a CMR. The control group included non-MTM enrollees who met MTM eligibility criteria but did not receive a CMR. Propensity score matching was utilized to ensure that the treatment and control groups contained patients with comparable characteristics [27, 28]. The propensity score represented the predicted probability of each individual receiving a CMR and was estimated by a logistic regression which accounted for all patient and community characteristics. Individuals in the control and treatment groups were then matched in a 3:1 ratio using the nearest neighbor propensity score without replacement [27, 28]. Finally, the propensity-score-matched treatment and control group members from each study year were pooled to form the final study sample.

The MTM eligibility criteria used by this study were based on the CMS guidelines and MTM program practices [11, 29, 30]. In general, Medicare Part D enrollees were deemed eligible for MTM services by Part D plans if the following three conditions were met: (1) had at least two to three chronic conditions; (2) had at least two to eight Part D covered medications; and (3) were likely to have minimum medications costs of $3138 in 2015, $3507 in 2016, and $3919 in 2017 [11, 29–31]. To account for the representative MTM eligibility thresholds in the analysis, the mode values of three chronic conditions and eight covered medications were analyzed for each study year [16, 32, 33]. A list of 25 chronic conditions was used to identify the number of chronic conditions a patient had [31].

A binary outcome variable was constructed to measure nonadherence to statin medications (nonadherent = 1; adherent = 0). While there are several hyperlipidemia medications available, statins were analyzed for this study since statins are the most widely used medications for hyperlipidemia treatment. Nonadherence was measured in terms of the proportion of days covered (PDC), in the same manner as the adherence measure for statin medications developed by the PQA and adopted by the CMS Star Ratings [34]. If the PDC was less than 80% for the statin medication received, the individual was considered nonadherent. Since the service date of the CMR receipt was available, the PDC was measured based on all prescriptions received after the CMR receipt date in the treatment group. For CMR non-recipients, the prescription records for the entire year were used to measure the outcome.

The conceptual framework for this study was the Gelberg-Andersen’s Behavioral Model for Vulnerable Populations [35]. The individual- and community-level characteristics were classified as predisposing, enabling, and need factors based on their relationship to prescription utilization [35]. Predisposing factors refer to patient characteristics that predetermine patients’ utilization of medications. For this study, individual-level predisposing factors were age, gender, and race/ethnicity. The community-level predisposing factors were the proportion of married-couple families, the proportion of the population with high school or higher education, income per capita, and the proportion of the uninsured population. The racial/ethnic groups included were Whites, Blacks, Hispanics, Asians/Pacific Islanders (Asians), and Others. The enabling factors in this study were community-level characteristics that represent the accessibility of healthcare services. These included metropolitan statistical area (MSA), health professional shortage area (HPSA), and census regions. Finally, the need factors are characteristics that represent an individual’s perceived or actual health status. For this study, the need factor was a risk adjustment summary score that indicates the expected healthcare expenses of the individual in relation to the average Medicare beneficiary [36].

Differences in characteristics were compared between CMR recipients and non-recipients by analyzing continuous variables with *t*-tests and categorical variables with Chi-squared tests. Chi-squared tests were conducted to examine the differences in the proportions of statin nonadherence across racial/ethnic groups between CMR recipients and non-recipients.

Multivariate logistic regression analyses were carried out in two stages. First, an adjusted regression was run separately for each study group to examine the factors affecting the likelihood of nonadherence, particularly the association between the outcome and each minority race dummy variable (Blacks, Hispanics, Asians, and Others) in comparison to Whites. Then, a DID analysis was performed using the same adjusted regression model and interaction terms between CMR receipt and race/ethnicity dummy variables with Whites as the reference group. The odds ratio (OR) of the interaction terms represented the effect of receiving a CMR on racial/ethnic disparity between minority groups and Whites. Specifically, receiving a CMR would be associated with reduced racial/ethnic disparities in the likelihood of statin medication nonadherence if the OR is negative.

Because multiple years of data were pooled for the analyses, some Medicare beneficiaries were likely to appear in more than one year if they met the inclusion criteria for multiple years. Robust standard errors were therefore used to account for potential correlations between the outcomes of a single beneficiary across different years. In addition, because community-level covariates were used, standard errors were clustered at the county level to account for possible correlations within a county. All statistical analyses were performed using SAS®9.4. This study was approved by the Institutional Review Board (approval number: #20-07197-XM) at the corresponding author’s institution.

## Results

The final study sample of 623,400 Medicare beneficiaries included 155,850 (25%) CMR recipients and 467,550 (75%) CMR non-recipients. Before propensity score matching, differences in patient characteristics between the two study groups were significant for age (*p* < 0.0001), race/ethnicity (*p* < 0.0001), per capita income (*p* < 0.0001), the proportion of uninsured population (*p* = 0.0333), MSA (*p* = 0.0488), distribution across geographic regions (*p* < 0.0001), and risk adjustment summary score (*p* < 0.0001). After matching, patient characteristics were similar for the two study groups, except CMR recipients were slightly younger (*p* = 0.0002). Each racial/ethnic group was represented in equal proportions in both study groups with 75.75% Whites, 10.40% Blacks, 8.63% Hispanics, 3.63% Asians, and 1.58% Others (Table 1).

**Table 1:** Characteristics among recipients and non-recipients of comprehensive medication review

Characteristics	Before Matching				After Matching			
	CMR Recipients ***n*** = 169,705		CMR Non-Recipients ***n*** = 776,670		CMR Recipients ***n*** = 155,850		CMR Non-Recipients ***n*** = 467,550	
	Number	%	Number	%	Number	%	Number	%
**Predisposing Factors**								
Age, mean (SD)**	79.10 (7.21)		80.82 (7.59)		79.59 (7.14)		79.75 (7.44)	
Male	60,417	35.60	280,854	36.16	56,393	36.18	169,179	36.18
Race/Ethnicity*								
Non-Hispanic Whites	120,141	70.79	583,401	75.12	118,064	75.75	354,192	75.75
Blacks	21,564	12.71	77,253	9.95	16,206	10.40	48,618	10.40
Hispanics	19,770	11.65	66,571	8.57	13,446	8.63	40,338	8.63
Asians/Pacific Islanders	5737	3.38	36,617	4.71	5664	3.63	16,992	3.63
Others	2493	1.47	12,828	1.65	2470	1.58	7410	1.58
Proportion of Married-Couple Families, mean (SD)^a^	0.72 (0.08)		0.72 (0.07)		0.72 (0.07)		0.72 (0.07)	
Proportion of Education ≥ High School, mean (SD)^a^	0.86 (0.06)		0.86 (0.06)		0.87 (0.06)		0.87 (0.06)	
Per Capita Income (in $1000), mean (SD)^a^*	49.18 (17.75)		50.58 (18.03)		49.34 (18.03)		49.60 (16.77)	
Proportion of No Insurance^a^*	0.11 (0.05)		0.11 (0.05)		0.11 (0.05)		0.11 (0.05)	
**Enabling Factors**								
Metropolitan Statistical Area^a^*	139,623	82.27	644,476	82.98	126,871	81.41	381,649	81.63
Health Professional Shortage Area^a^	155,390	91.56	712,252	91.71	142,052	91.15	426,653	91.25
Census Regions^a^*								
Northeast	39,502	23.28	183,280	23.60	35,233	22.61	106,521	22.78
Midwest	38,903	22.92	149,457	19.24	34,996	22.45	103,366	22.11
South	67,118	39.55	317,480	40.88	62,874	40.34	189,426	40.51
West	24,182	14.25	126,453	16.28	22,747	14.60	68,237	14.59
**Need Factor**								
Risk Adjustment Summary Score, mean (SD)*	2.48 (1.69)		2.73 (1.52)		2.59 (1.70)		2.59 (1.43)	

*Abbreviations*: *CMR* Comprehensive Medication Review, *SD* Standard deviation

^a^Community-level factor; * before propensity score matching, the difference between CMR recipients and non-recipients was significant (*p* < 0.05); ** before and after propensity score matching, the difference between CMR recipients and non-recipients was significant (*p* < 0.01)

**Fig. 1:**
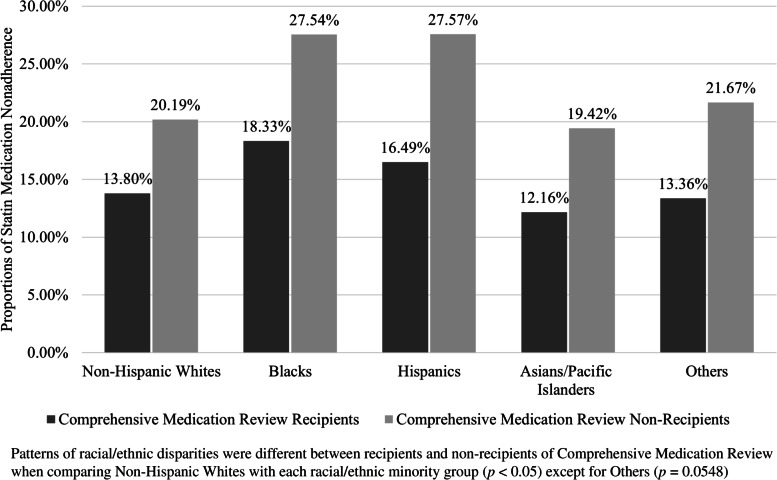
Nonadherence to statin medications across racial/ethnic groups among recipients and non-recipients of comprehensive medication review

Within each of the two study groups, differences in the proportions of statin medication nonadherence across racial/ethnic groups showed a similar pattern in which both Blacks and Hispanics had higher proportions of nonadherence than Whites (Fig. 1). However, the gap between Whites and these two minority groups was smaller among CMR recipients than non-recipients. Specifically, the difference in the proportions of nonadherence between Blacks and Whites was 4.53% (18.33% vs. 13.80%) for CMR recipients and 7.35% (27.54% vs. 20.19%) for non-recipients. Similarly, the gap in the proportions of nonadherence between Hispanics and Whites was 2.69% (16.49% vs. 13.80%) for CMR recipients and 7.38% (27.57% vs. 20.19%) for non-recipients. For both CMR recipients and non-recipients, Asians had a lower proportion of nonadherence compared to Whites. Patterns of racial/ethnic disparities between CMR and non-CMR recipients were found to be significant in the unadjusted analysis for each racial/ethnic minority group (*p* < 0.05) except for Others (*p* = 0.0548).

Table 2 presents the results of the multivariate analyses that separately examined racial/ethnic disparities in statin medication nonadherence among CMR recipients and non-recipients. Blacks and Hispanics had higher odds of nonadherence than Whites among CMR recipients and non-recipients; Others had higher odds of nonadherence than Whites among CMR non-recipients.

**Table 2:** Racial/ethnic disparities in nonadherence to statin medications among study groups

	CMR recipients *n* = 155,850		CMR non-recipients *n* = 467,550	
	Odds Ratio	95% Confidence Interval	Odds Ratio	95% Confidence Interval
**Predisposing Factors**				
Race/Ethnicity				
Blacks	1.29	1.23 – 1.36	1.24	1.21 – 1.28
Hispanics	1.21	1.12 – 1.31	1.28	1.21 – 1.35
Asians/Pacific Islanders	1.00	0.90 – 1.12	1.03	0.90 – 1.16
Others	1.00	0.86 – 1.17	1.11	1.01 – 1.22
Age	1.00	1.00 – 1.00	1.00	1.00 – 1.00
Male	0.87	0.84 – 0.90	0.90	0.89 – 0.91
Proportion of Married-Couple Families^a^	0.74	0.56 – 0.98	0.68	0.55 – 0.84
Proportion of Education ≥ High School^a^	1.57	0.91 – 2.72	1.22	0.82 – 1.83
Per Capita Income (in $1000)^a^	1.00	1.00 – 1.00	1.00	1.00 – 1.00
Proportion of No Insurance^a^	2.24	1.34 – 3.75	3.39	2.46 – 4.66
Year 2016	1.01	0.97 – 1.04	0.93	0.91 – 0.95
Year 2017	0.82	0.79 – 0.85	0.79	0.77 – 0.81
**Enabling Factors**				
Metropolitan Statistical Area^a^	0.98	0.94 – 1.03	1.05	1.02 – 1.08
Health Professional Shortage Area^a^	1.02	0.96 – 1.07	1.01	0.97 – 1.05
Census Regions^a^				
Midwest	1.00	0.95 – 1.06	1.02	0.98 – 1.08
South	1.12	1.06 – 1.19	1.15	1.11 – 1.20
West	0.96	0.88 – 1.06	1.02	0.94 – 1.10
**Need Factor**				
Risk Adjustment Summary Score	1.19	1.18 – 1.20	1.21	1.20 – 1.22

Reference groups for categorical variables: Non-Hispanic Whites, female, year 2015, non-Metropolitan Statistical Area, non-Health Professional Shortage Area, and Northeast region. Robust standard errors clustered at county level

*Abbreviation*: *CMR* Comprehensive Medication Review

^a^Community-level factors

Other patient and community characteristics also exhibited significant association with statin medication nonadherence. Among both CMR recipients and non-recipients, being male and living in a county with a higher proportion of married-couple families were associated with lower odds of nonadherence. In contrast, living in a county with a higher proportion of the uninsured population, living in the South (compared to Northeast region), and having a higher risk adjustment summary score were each associated with an increased odds of nonadherence.

Results from the DID analysis were reported in Table 3. The ORs for the interaction terms for CMR and Hispanics, Asians, and Others were negative, but only the OR for the interaction term between CMR and Hispanics was significant (OR = 0.89; 95% Confidence Interval or CI = 0.85-0.94).

**Table 3:** Effects of comprehensive medication review on racial/ethnic disparities in nonadherence to statin medications

	Coefficient Estimate	Standard Error	Odds Ratio	95% Confidence Interval	***P*** value
**Predisposing Factors**					
Race/Ethnicity					
Blacks	0.22	0.02	1.25	1.21–1.29	< 0.0001
Hispanics	0.26	0.03	1.29	1.23–1.36	< 0.0001
Asians/Pacific Islanders	0.03	0.07	1.03	0.91–1.18	0.63
Others	0.10	0.05	1.11	1.01–1.22	0.03
CMR receipt	−0.49	0.01	0.61	0.60–0.63	< 0.0001
CMR × Blacks	0.01	0.02	1.01	0.97–1.06	0.58
CMR × Hispanics	−0.11	0.03	0.89	0.85–0.94	< 0.0001
CMR × Asians/Pacific Islanders	−0.06	0.05	0.94	0.85–1.05	0.27
CMR × Others	−0.11	0.07	0.89	0.77–1.03	0.12
Age	−0.002	0.0006	1.00	1.00–1.00	0.0001
Male	−0.11	0.01	0.89	0.88–0.91	< 0.0001
Proportion of Married-Couple Families^a^	−0.37	0.10	0.69	0.57–0.83	< 0.0001
Proportion of Education ≥ High School^a^	0.25	0.20	1.28	0.87–1.90	0.21
Per Capita Income (in $1000)^a^	< 0.0001	0.0004	1.00	1.00–1.00	0.93
Proportion of No Insurance^a^	1.13	0.15	3.11	2.33–4.15	< 0.0001
Year 2016	−0.06	0.01	0.94	0.93–0.96	< 0.0001
Year 2017	−0.23	0.01	0.80	0.78–0.81	< 0.0001
**Enabling Factors**					
Metropolitan Statistical Area^a^	0.04	0.01	1.04	1.01–1.07	0.01
Health Professional Shortage Area^a^	0.01	0.02	1.01	0.98–1.04	0.55
Census Regions^a^					
Midwest	0.02	0.02	1.02	0.98–1.07	0.38
South	0.14	0.02	1.15	1.11–1.19	< 0.0001
West	0.01	0.04	1.01	0.94–1.09	0.81
**Need Factor**					
Risk Adjustment Summary Score	0.19	0.004	1.21	1.20–1.21	< 0.0001

Reference groups for categorical variables: Non-Hispanic Whites, CMR non-recipients, female, year 2015, non-Metropolitan Statistical Area, non-Health Professional Shortage Area, and Northeast region

*Abbreviation*: *CMR* Comprehensive Medication Review

^a^Community-level factors

This suggests that the disparities between Hispanics and Whites in the odds of statin medication nonadherence was 11% lower among CMR recipients compared to non-recipients. In the results from the DID analysis, similar patient and community characteristics had a significant association with statin nonadherence as in Table 2.

## Discussion

This study used the Medicare data from 2015 to 2017 to evaluate the effect of CMRs on racial/ethnic disparities in statin medication nonadherence among Medicare beneficiaries aged 65 years or older with AD. It was found that the disparities between Whites and Hispanics experienced a higher reduction among CMR recipients relative to CMR non-recipients. The finding of higher disparity reduction between Whites and Hispanics among CMR recipients supports that CMRs can improve medication adherence among Hispanics in the Medicare population with AD. However, since disparities persist between Whites and Hispanics among CMR recipients, barriers to adherence persist among Hispanics.

As previously mentioned, AD is a chronic condition that only a small percentage of MTM programs target [16, 17, 32]. These findings from this study on the benefits of CMRs among the study population with AD suggest that improving plans’ MTM program to better target AD is crucial. This is especially true because the completion rates of CMRs are increasing, but the rates remain low, especially among stand-alone Part D plans. According to a Pharmacy Quality Solutions Star Ratings report, the average CMR completion rate for Medicare Advantage plans increased from 77% in 2020 to 81% in 2021, while the average CMR completion rate for stand-alone Part D plans increased from 44% in 2020 to 49% in 2021 [37]. The finding of this study further supports the need to continue increasing CMR completion rates.

Racial/ethnic disparities among patients with AD are apparent and prevalent. For example, Hispanics and Blacks have an increased risk for AD development, and both minority groups have remained underrepresented in research [1, 38]. Although this study found racial/ethnic disparity reduction between Hispanics and Whites, this study did not detect significant effects of CMRs on disparities between Blacks and Whites. A few explanations are plausible for such a pattern. One possible reason may be that this study failed to account for the severity of AD. Previous studies have found that the Blacks are at higher risk for more severe AD symptoms, more likely to have the apolipoprotein E isoform associated with AD development, and more likely to have dementia caused by mixed pathology [39, 40]. Blacks may have reduced memory function due to more progressed AD and may require more intense interventions before experiencing improved medication adherence than a standard CMR.

Another plausible reason that disparity reduction was not detected between Blacks and Whites may lie in the fact that the medications measured for nonadherence in this study, statins, are not the only medications used for hyperlipidemia treatment. While statins are the mainstay of hyperlipidemia treatment, there are alternative medication options, such as ezetimibe and PCSK9 inhibitors, for those who cannot tolerate statins [41]. It has been shown that the Blacks have decreased statin usage and statin adherence rates compared to Whites, likely because of an overall decreased perception of statin safety among the Black population [42]. Therefore, this may have obstructed the disparity reduction between Blacks and Whites in this study.

Furthermore, Hispanics may more readily accept provider advice compared to other races/ethnicities [43]. For instance, Hispanics have decreased hospitalization and emergency department visitations after diagnosis of AD-related dementia compared to prior to diagnosis [43]. This may indicate that Hispanics are more proactive in using their newfound health knowledge to prevent further complications. Therefore, in the context of this study, Hispanics may be more attentive to the medication adherence advice given by the CMR providers than other minority races/ethnicities.

The study findings also revealed that some community-level factors, such as living in a county with a higher proportion of uninsured population and living in the South, were associated with medication nonadherence. It has been documented that uninsured adults, compared to those insured, have fewer healthcare encounters and are less often treated for hyperlipidemia [44]. The Southern region, compared to other regions, has been demonstrated to have worse statin adherence among the Medicare population [45]. Further, individuals with no insurance and those that live in the South have higher disease burden and are frequently racial/ethnic minorities [44, 46]. Therefore, living in a community with a high proportion of uninsured individuals and living in the South may represent barriers to medication adherence and contribute to worse racial/ethnic health disparities. Efforts to improve medication adherence should include targeting such underserved populations and areas.

The study finding should be considered with a few caveats. First, there is limited information on individual-level characteristics in the Medicare data. Consequently, significant number of variables in this study represent community characteristics at the county level, which may imprecisely represent characteristics of individual Medicare beneficiaries. Secondly, races/ethnicities other than Blacks and Whites are under-identified in Medicare enrollment data [47]. Thus, potential racial/ethnic disparities other than between Blacks and Whites may be underestimated in this study. Another limitation is that some patients may be included in the study sample for more than one year. As a result, there might be correlations between the outcomes for the same individuals over time. However, robust standard errors were applied in this study to control for these potential correlations. Additionally, the clinical reasons for individuals being prescribed a statin or taking different statin doses were not explored. Lastly, the outcome measure of nonadherence was based on PDC. Although this is a validated measure used in research and adopted by CMS for Star Ratings, this measure was based on records of prescription fills but not prescription intake. Even with these limitations, the study made significant contribution by examining the effect of CMRs on racial/ethnic disparities among the AD population.

## Conclusions

CMRs were found to be associated with reduced disparities in statin medication nonadherence between Hispanic Medicare beneficiaries with AD and their Whites counterparts. This study has enhanced the existing knowledge about the benefits of CMRs, specifically among the AD population. Utilizing CMRs to improve medication adherence among the study population offers a potential solution for racial/ethnic disparities among patients with AD. Therefore, there is a need to increase the number of MTM programs that target AD and to improve CMR completion rates among Medicare beneficiaries. Such strategies can expand the population benefiting from CMRs and further reduce racial/ethnic disparities. Future research is warranted to explore the effects of CMRs and other MTM services on racial/ethnic disparities in other chronic conditions and with other outcome measures.

## Data Availability

The data analyzed for this study are available from the CMS Virtual Research Data Center with strict access restrictions.

## Abbreviations

AD: Alzheimer’s disease
AHRF: Area Health Resources Files
Asians: Asians/Pacific Islanders
CMR: Comprehensive medication review
CI: Confidence interval
CMS: Centers for Medicare and Medicaid Services
DID: Difference-in-Differences
HPSA: Health professional shortage area
ICD-9: International Classification of Diseases version 9
ICD-10: International Classification of Diseases version 10
MBSF: Master Beneficiary Summary File
MSA: Metropolitan statistical area
MTM: Medication therapy management
OR: Odds ratio
PDC: Proportion of days covered
PDE: Part D Event
PQA: Pharmacy Quality Alliance
Whites: Non-Hispanic Whites

## References

[1] 2020 Alzheimer’s disease facts and figures. Alzheimers Dement. 2020;16(3):391–460. 10.1002/alz.12068. 10.1002/alz.12068 32157811

[2] Centers for Disease Control and Prevention. CDC wonder: about underlying cause of death, 1999-2019. https://wonder.cdc.gov/UCD-ICD10.html. Accessed 16 Aug 2021.

[3] United States Census Bureau (2020). 2017 national population projections tables: main series.

[4] Hebert LE, Weuve J, Scherr PA, Evans DA (2013). Alzheimer disease in the United States (2010-2050) estimated using the 2010 census. Neurology.

[5] Stefanacci RG (2011). The costs of Alzheimer’s disease and the value of effective therapies. Am J Manag Care.

[6] Hofman A, Ott A, Breteler MM, Bots ML, Slooter AJ, van Harskamp F (1997). Atherosclerosis, apolipoprotein E, and prevalence of dementia and Alzheimer’s disease in the Rotterdam study. Lancet.

[7] Zhou Z, Liang Y, Zhang X, Xu J, Lin J, Zhang R (2020). Low-density lipoprotein cholesterol and Alzheimer’s disease: a systematic review and meta-analysis. Front Aging Neurosci.

[8] Agarwal M, Khan S (2020). Plasma lipids as biomarkers for Alzheimer’s disease: a systematic review. Cureus.

[9] Sáiz-Vazquez O, Puente-Martínez A, Ubillos-Landa S, Pacheco-Bonrostro J, Santabárbara J (2020). Cholesterol and Alzheimer’s disease risk: a meta-meta-analysis. Brain Sci.

[10] Department of Health & Human Services, Centers for Medicare & Medicaid Services (2014). Medicare program; contract year 2015 policy and technical changes to the Medicare advantage and the Medicare prescription drug benefit programs.

[11] Center for Medicare (2014). CY 2015 medication therapy management program guidance and submission instructions.

[12] Medication Therapy Management. Centers for Medicare & Medicaid Services. https://www.cms.gov/Medicare/Prescription-Drug-Coverage/PrescriptionDrugCovContra/MTM. Accessed 16 Aug 2021.

[13] Ferries E, Dye JT, Hall B, Ndehi L, Schwab P, Vaccaro J (2019). Comparison of Medication Therapy Management services and their effects on health care utilization and medication adherence. J Manag Care Spec Pharm.

[14] Erku DA, Ayele AA, Mekuria AB, Belachew SA, Hailemeskel B, Tegegn HG (2017). The impact of pharmacist-led medication therapy management on medication adherence in patients with type 2 diabetes mellitus: a randomized controlled study. Pharm Pract (Granada).

[15] Gray C, Cooke CE, Brandt N (2019). Evolution of the Medicare Part D Medication Therapy Management program from inception in 2006 to the present. Am Health Drug Benefits.

[16] Centers for Medicare & Medicaid Services (2017). 2017 Medicare part D medication therapy management (MTM) programs.

[17] Centers for Medicare & Medicaid Services (2019). 2019 Medicare part D medication therapy management (MTM) programs.

[18] Wang J, Qiao Y, Spivey CA, Li C, Clark C, Deng Y (2016). Disparity implications of proposed 2015 Medicare eligibility criteria for medication therapy management services. J Pharm Health Serv Res.

[19] Health Resources & Services Administration Data Warehouse. Area health resources files. https://data.hrsa.gov/topics/health-workforce/ahrf. Accessed 16 Aug 2021.

[20] Research Data Assistance Center. Data file search. https://www.resdac.org/cms-data?tid_1%5B%5D=1. Accessed 16 Aug 2021.

[21] Research Data Assistance Center. Medicare Master Beneficiary Summary File (MBSF) Base. https://www.resdac.org/cms-data/files/mbsf-base. Accessed 16 Aug 2021.

[22] Chronic Conditions Data Warehouse. Condition categories. https://www2.ccwdata.org/web/guest/condition-categories. Accessed 16 Aug 2021.

[23] Research Data Assistance Center. Part D event (PDE) file. https://www.resdac.org/cms-data/files/pde. Accessed 16 Aug 2021.

[24] Research Data Assistance Center. Part D medication therapy management data file. https://www.resdac.org/cms-data/files/part-d-mtm. Accessed 16 Aug 2021.

[25] Health Insurance Marketplace. 2017 quality rating system measure technical specifications. In: Quality initiatives - general information. Centers for Medicare & Medicaid Services; 2016. https://www.cms.gov/Medicare/Quality-Initiatives-Patient-Assessment-Instruments/QualityInitiativesGenInfo/Downloads/2017_QRS-Measure_Technical_Specifications.pdf. Accessed 16 Aug 2021.

[26] Health Insurance Exchange. 2018 quality rating system measure technical specifications. In: quality initiatives – general information. Centers for Medicare & Medicaid Services; 2017. https://www.cms.gov/Medicare/Quality-Initiatives-Patient-Assessment-Instruments/QualityInitiativesGenInfo/Downloads/Revised_QRS-2018-Measure-Tech-Specs_20170929_508.pdf. Accessed 16 Aug 2021.

[27] Rosenbaum PR, Rubin DB (1983). The central role of the propensity score in observational studies for causal effects. Biometrika.

[28] Austin PC (2014). A comparison of 12 algorithms for matching on the propensity score. Stat Med.

[29] Center for Medicare. CY 2016 medication therapy management program guidance and submission instructions. Department of Health & Human Services, Centers for Medicare & Medicaid Services; 2015. https://www.cms.gov/Medicare/Prescription-Drug-Coverage/PrescriptionDrugCovContra/Downloads/Memo-Contract-Year-2016-Medication-Therapy-Management-MTM-Program-Submission-v-040715.pdf. Accessed 16 Aug 2021.

[30] Center for Medicare. CY 2017 medication therapy management program guidance and submission instructions. Department of Health & Human Services, Centers for Medicare & Medicaid Services; 2016. https://www.cms.gov/Medicare/Prescription-Drug-Coverage/PrescriptionDrugCovContra/Downloads/Memo-Contract-Year-2017-Medication-Therapy-Management-MTM-Program-Submission-v-040816.pdf. Accessed 16 Aug 2021.

[31] Daniel GW, Malone DC (2007). Characteristics of older adults who met the annual prescription drug expenditure threshold for Medicare medication therapy management programs. J Manag Care Pharm.

[32] Centers for Medicare & Medicaid Services (2015). 2015 medicare part D medication therapy management (MTM) programs.

[33] Centers for Medicare & Medicaid Services (2016). 2016 Medicare part D medication therapy management (MTM) programs.

[34] Centers for Medicare & Medicaid Services. Part C and D performance data. https://www.cms.gov/Medicare/Prescription-Drug-Coverage/PrescriptionDrugCovGenIn/PerformanceData. Accessed 16 Aug 2021.

[35] Gelberg L, Andersen RM, Leake BD (2000). The behavioral model for vulnerable populations: application to medical care use and outcomes for homeless people. Health Serv Res.

[36] Centers for Medicare & Medicaid Services. Chapter 7 - Risk adjustment. In: Medicare managed care manual. https://www.cms.gov/Regulations-and-Guidance/Guidance/Manuals/Downloads/mc86c07.pdf. Accessed 16 Aug 2021.

[37] Pharmacy Quality Solutions. PQS summary of 2021 Medicare part C and D star ratings technical notes. https://www.pharmacyquality.com/wp-content/uploads/2020/10/PQSMedicareStarRatingsUpdate2021NEW.pdf. Accessed 16 Aug 2021.

[38] Chen C, Zissimopoulos JM (2018). Racial and ethnic differences in trends in dementia prevalence and risk factors in the United States. Alzheimers Dement (N Y).

[39] Livney MG, Clark CM, Karlawish JH, Cartmell S, Negrón M, Nuñez J (2011). Ethnoracial differences in the clinical characteristics of Alzheimer’s disease at initial presentation at an urban Alzheimer’s disease center. Am J Geriatr Psychiatry.

[40] Barnes LL, Leurgans S, Aggarwal NT, Shah RC, Arvanitakis Z, James BD (2015). Mixed pathology is more likely in black than white decedents with Alzheimer dementia. Neurology.

[41] Diaconu CC, Iorga RA, Furtunescu F, Katsiki N, Stoian AP, Rizzo M (2021). Statin intolerance: new data and further options for treatment. Curr Opin Cardiol.

[42] Nanna MG, Navar AM, Zakroysky P, Xiang Q, Goldberg AC, Robinson J (2018). Association of patient perceptions of cardiovascular risk and beliefs on statin drugs with racial differences in statin use: insights from the patient and provider assessment of lipid management registry. JAMA Cardiol.

[43] Downer B, Al Snih S, Chou L-N, Kuo Y-F, Raji M, Markides KS (2021). Changes in health care use by Mexican American Medicare beneficiaries before and after a diagnosis of dementia. Newman AB, editor. J Gerontol Ser A Biol Med Sci.

[44] Egan BM, Li J, Sarasua SM, Davis RA, Fiscella KA, Tobin JN (2017). Cholesterol control among uninsured adults did not improve from 2001-2004 to 2009-2012 as disparities with both publicly and privately insured adults doubled. J Am Heart Assoc.

[45] Couto JE, Panchal JM, Lal LS, Bunz TJ, Maesner JE, O’Brien T (2014). Geographic variation in medication adherence in commercial and Medicare part D populations. J Manag Care Spec Pharm.

[46] Miller CE, Vasan RS (2021). The southern rural health and mortality penalty: a review of regional health inequities in the United States. Soc Sci Med.

[47] Zaslavsky AM, Ayanian JZ, Zaborski LB (2012). The validity of race and ethnicity in enrollment data for Medicare beneficiaries. Health Serv Res.

